# Complete mitochondrial genome of *Allognosta vagans* (Diptera, Stratiomyidae)

**DOI:** 10.1080/23802359.2017.1357450

**Published:** 2017-07-26

**Authors:** Qingxia Zhou, Shuangmei Ding, Xin Li, Tingting Zhang, Ding Yang

**Affiliations:** a College of Plant Protection, China Agricultural University, Beijing, China;; b Department of Entomology, Shandong Agricultural University, Taian, China

**Keywords:** Stratiomyidae, phylogeny, mitogenome, *Allognosta vagans*

## Abstract

The complete mitochondrial genome (mitogenome) of *Allognosta vagans* (Loew, 1873) has been reported in this study. This is the first sequenced mitogenome of the subfamily Beridinae. The genome is 15,982 bp in length, including 13 protein-coding genes, 2 ribosomal RNAs, 22 transfer RNAs, and a partial sequence of the AT-rich region, and the AT-rich region contains several characteristic repeated sequences. In addition, the nucleotide composition of the coding region was 38.8% of A, 38.7% of T, 9.4% of C, 13.1% of G, 77.5% of A + T content. So far, five complete mitochondrial genome data of related species are available in our lab, all of them are used in Maximum Likelihood and Neighbour-Join analyses. The result supported that Xylomyidae and Stratiomyidae are sister group.

The superfamily Stratiomyidae is one of the primitive groups in lower Brachycera. It is divided into two families, Xylomyidae and Stratiomyidae (Sinclair 1992; Sinclair et al. 1994; Wiegmann et al. 2000). Stratiomyidae is a highly diversified group with 382 genera and over 3000 known species all over the world, and 55 genera and 346 known species in China. Some species of Stratiomyidae can harm crops, but also some can be used as fodder, therefore many biological studies were worked on them (Yang et al. 2014). However, there are few mitochondrial genome data of Stratiomyidae. Complete mitochondrial genomes of its relative species *Dialy*sis sp., *Trichophthalma punctata*, *Ptecticus aurifer*, and *Xylomya moiwana* are available in our laboratory. In addition, we sequenced the complete mitochondrial genome of *Allognosta vaga*ns (Loew, 1873), the first representative of subfamily Beridinae for the further research.

Specimens of *Allognosta vagans* were collected in Tianmu Mountain, Linan City, Zhejiang Province, China by Tingting Zhang, and also identified by Tingting Zhang. Specimens are deposited in the Entomological Museum of China Agricultural University, Beijing.

The genomic DNA was extracted from adult’s muscle tissues of the thorax using the DNeasy DNA Extraction kit (TIANGEN, Beijing, China), and sequenced under the next generation sequence technology.

The complete mitochondrial genome of *Allognosta vagans* contains 22 transfer RNA genes, 13 protein-coding genes (PCG), 2 ribosomal RNA genes and non-coding control region, which were similar with related reports before (Kang et al. 2014; Li et al. 2016; Wang et al. 2016a, 2016b, 2016c).

The completed mitochondrial genome of *Allognosta vagans* is 15,982 bp in length, and the nucleotide composition of coding region was 38.8% of A, 38.7% of T, 9.4% of C, 13.1% of G, 77.5% of A + T content. The codon ATG was the most popular start codon which was shared with ATP6, COX2, COX3, CYTB, ND4, ND4L, and start codon ATT was shared with ND2, ND3, ND5, ND6. Particularly, the ATP8 begins with codon ATC, the COX1 begins with codon TCG, and the ND1 begins with codon TTG. The conservative stop codon TAA was shared with ATP6, ATP8, COX1, COX2, COX3, CYTB, ND2, ND4L, ND6, and the stop codon T was shared with ND1, ND4, ND5, while the gene ND3 was ended with stop codon TAG.

Based on 13 PCGs data among seven species including *Chironomus tepperi* from Chironomidae and *Tipula abdominalis* from Tipulidae as outgroups, we conducted a phylogenetic analysis employing the Neighbour-Join method (NJ) implemented in Mega 7 (Kumar et al. 2016) and Maximum Likelihood (ML) method in RaxML 7.0.3 (Stamatakis 2006). As NJ tree and ML tree have the complete same topology, only one tree labelled by two kinds of support values was given (Figure 1). In NJ and ML analyses, the monophyly of both Xylomyidae and Stratiomyidae was consistently supported, and Xylomyidae is considered as the sister group of Stratiomyidae.

**Figure 1.**
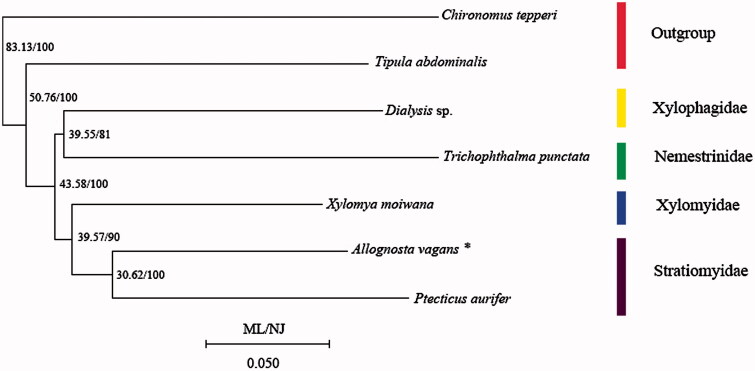
Phylogenetic tree among seven species which consist of five lower Brachycera species and two outgroups including Chironomidae and Tipulidae (*data sequenced in this study).
